# Constructing an emergency preparedness evaluation index system for public use during major emerging infectious disease outbreaks: a Delphi study

**DOI:** 10.1186/s12889-023-15980-6

**Published:** 2023-06-08

**Authors:** Wei Wei, Yubei Liu, Na Zhou, Min Tian, Longsheng Xie, Roger Watson, Fengling Dai, Yanhua Chen, Weili Hu

**Affiliations:** 1 School of Nursing, Southwest Medical University, 1 Xianglin Road, Luzhou, China; 2 Department of Orthopedics, The Affiliated Hospital of Southwest Medical University, 25 Taiping Street, Luzhou, China; 3 Department of Nephrology, The Affiliated Hospital of Southwest Medical University, 25 Taiping Street, Luzhou, China; 4 Health and Social Care Faculty, University of Hull, Cottingham Road, Hull, HU6 7RX USA; 5 Department of Science and Technology, Southwest Medical University, 1 Xianglin Road, Luzhou, China; 6 Department of Nursing, The Affiliated Hospital of Southwest Medical University, 25 Taiping Street, Luzhou, China; 7 College of Humanities and Management, Southwest Medical University, 1 Xianglin Road, Luzhou, China

**Keywords:** Delphi technique, Major emerging infectious diseases, Emergency preparedness, Evaluation research

## Abstract

**Background:**

The major emerging infectious diseases (MEIDs) have occurred frequently and become increasingly serious in the world. Sufficient personal emergency preparedness is critical for the general people in efficiently responding to and recovering from MEIDs. Nevertheless, few specific indicators are available for assessing the individual emergency preparedness of the general public during these periods. Therefore, the aim of this study was to construct an index system for comprehensively evaluating the personal emergency preparedness of the public regarding MEIDs.

**Methods:**

Based on the global national-level emergency preparedness index framework and a literature review, a preliminary index system was constructed. From June 2022 to September 2022, a panel of 20 experts from nine provinces and municipalities across multiple research areas participated in this Delphi study. They rated the importance of pre-defined indicators using a five-point Likert scale and provided their qualitative comments. According to the feedback of each round of experts, the indicators of the evaluation index system were revised.

**Results:**

After two rounds of expert consultation the evaluation index system reached a consensus, containing five first-level indicators, cooperating with prevention and control work, improving emergency response capacity, securing supplies and equipment, preparing economic resources, maintaining physical and mental health with affiliated 20 s-level indicators and 53 third-level indicators. The expert authority coefficient of consultation was 0.88 and 0.90. The Kendall’s coefficient of concordance of expert consultations was 0.294 and 0.322, respectively. The differences were statistically significant (*P* < 0.05).

**Conclusion:**

A valid, reliable and scientific evaluation index system was established. This personal emergency preparedness index system, as a precursor form, will further lay the foundation for the formation of an assessment instrument. At the same time, it could provide a reference for future education and training of emergency preparedness for the general public.

**Supplementary Information:**

The online version contains supplementary material available at 10.1186/s12889-023-15980-6.

## Background

Since the 21st century, major emerging infectious diseases (MEIDs) have occurred frequently and become increasingly serious, against a background of globalization and economic and cultural exchange [1]. MEIDs such as Severe Acute Respiratory Syndrome (SARS), human avian influenza, influenza A (H1N1), avian influenza A (H7N9), Ebola hemorrhagic fever, the Middle East Respiratory Syndrome (MERS), and Corona Virus Disease 2019 (COVID-19) have appeared in recent years [2]. However, MEIDs have the characteristics of suddenness, high infectivity and uncertain prognosis, which seriously affect public health, social stability and economic development [3]. Countries across the world have realized that preventing and controlling MEIDs has become a significant public health issue [4, 5].

Emergency management cannot completely rely on the power of the government [6]. It requires the joint participation and close cooperation of multiple sectors of society. It is necessary for the public to have the facility to identify risks early, respond scientifically to disasters and rebuild after disasters. Although MEIDs usually appear suddenly, appropriate preparedness of the public can mitigate the harm caused by MEIDs [7]. The COVID-19 has provided a stark example of the need for emergency preparedness in outbreak preparedness [8]. Emergency preparedness of the general public for MEIDs is defined in this study as the capacity of individuals effectively to prevent, respond to, and recover from MEIDs, including the preparedness of emergency skills, legal compliance, avoiding secondary disasters, economic estimation, and somatopsychic health [9].

Although studies have been developed in recent years to evaluate the emergency preparedness of the national government, public health departments, and healthcare workers during MEIDs [10–12], few studies have focused in depth on emergency preparedness at the public level. Ou Yanling et al [13] developed an evaluation index system of residents health emergency literacy for major epidemics. The evaluation content of this index system was not sufficiently comprehensive because it mainly screened out indicators from the six areas of basic cognition, basic attitude, scientific knowledge, behavioral knowledge, cognitive skills and operational skills of major epidemic health emergency, and did not include comprehensive preparation for legal compliance, economic estimation, material reserves, physical and mental health. Heagele TN et al [14] constructed Household Emergency Preparedness Instrument, which had four dimensions: preparedness actions; communication plans; evacuation plans; and disaster supplies. The tool was highly reliable, but the results obtained were only on the competencies required in routine situations and did not address the competencies required during MEIDs. Therefore, this study intends to construct a standardized, comprehensive and practical emergency preparedness evaluation index system for public use during MEIDs.

## Methods

This study was approved by the Ethics Committee of the Affiliated Hospital of Southwest Medical University (NO. KY2022345). A Delphi study was conducted to explore experts’ attitude towards the evaluation index system. To explore the rationality of it, each expert was invited to rate the importance of a series of pre-defined indexes independently [15]. A modified recommendation for the Conducting and Reporting of Delphi studies (CREDES) was used to guide the study [16].

### Study design

The Delphi method is considered to be an established and adaptable research method for querying experts and ultimately transforming expert opinions into group consensus [17]. Meanwhile, Delphi study is regarded as a flexible research methodology to set goals, items, etc. Researchers can send questionnaires and communicate with experts online or offline, so they do not need to consider the geographical location of experts. The process of gathering experts’ advice is independent and anonymous, ensuring that the experts will not discuss or exchange views [18]. Through iterative multistage process, accurate and reliable data will eventually be obtained.

### Expert selection

Purposive sampling augmented by snowball recruitment was employed to deliberately select Delphi panel members from different regions and organizations in China [19], with the following inclusion and exclusion criteria: 1.inclusion criteria: (a) they engaged in public health management, infectious diseases prevention and control, disease control management, or psychology (those who have post-epidemic psychological counseling experience ); (b) their work experience over eight years; (c) they obtained the professional title qualification of intermediate or above; (d) they had bachelor’s degree or above; and (e) they voluntarily participate in this study and actively give feedback. 2. exclusion criteria: (a) they were reluctant to participate in this study due to personal reasons; (b) they had no practical experience of working with MEIDs. (c) they withdrew from the study while the study was ongoing. Finally, we recruited 20 experts according to the published recommendations [20].

### Construction an initial evaluation index system

We constructed an educational content framework for emergency preparedness of the public during MEIDs, which has been published [9]. Based on our previous studies, supplemented by literature review, a preliminary draft of the evaluation index system was constructed. The initial draft hinged on the global national-level emergency preparedness index framework [10], the emergency plan for public health emergencies [21] and the citizen health emergency literacy in China [22]. Furthermore, the initial evaluation index system included five first-level indicators, 21 s-level indicators and 55 third-level indicators (Appendix 1). The first-level indicators are the main dimension of the public emergency preparedness, and the core indicators. The second-level indicators are classified and defined according to the emergency preparedness contents contained under the first-level indicator. The third-level indicators are the specific evaluation indicators under the second-level indicator.

### Questionnaire design

The expert consultation questionnaire was composed of four parts. (1) Preface: this section briefly explained the research purpose, the content, the requirements for completing the questionnaire, etc. (2) General information of the experts: age, educational background, professional title, working years, research fields, etc. (3) Expert consultation form of the evaluation index system: this part was the main body of assessment in the consultation questionnaire, all the indicators were shown in this section. And the importance of each indicator was assessed by experts using the Likert five-level scoring method. Points were scored from five to one in order: very important = 5, important = 4, neutral = 3, unimportant = 2, and completely unimportant = 1. In addition, the experts could freely express their personal ideas and suggestions in this part to enrich the content for the evaluation index system [23]. (4) Expert familiarity with the content of the study and index judgment.

### Delphi consulting and feedback cycle

The researchers contacted the experts personally via emails, WeChat® or other means between June and September 2022, experts did not communicate with each other. The researchers sorted out the opinions, suggestions and feedback of each expert, and readjusted the consultation questionnaire for the second round for further confirm accuracy of the evaluation index system [24]. Additionally, the index inclusion criterion: the average importance score of each index evaluated by the expert panel > 3.5, the coefficient of variation < 2.5, and the full mark rate > 20% [25]. When some important indicators were not in the inclusion criterion, the research team needed to discuss whether the indicator was retained or deleted [26]. After two rounds of expert consultation, the evaluation index system reached a consensus.

### Data analysis

Excel 2010 and SPSS 25.0 software were used for statistical analysis. Frequency and percentage are used to describe the personal information of experts. The enthusiasm of experts was expressed by the response rate of the questionnaire. The authority coefficient (Cr) of the expert was the mean value of the expert’s judgment basis (Ca) and degree of familiarity (Cs) with the research content, based on the formula Cr = (Ca + Cs)/2. The degree of expert’s opinion dispersion was represented by the Kendall coefficient of concordance (Kendall’s W) and coefficient of variation (CV), also the importance of each index was described by mean ± standard deviation. *P* < 0.05 was considered to indicate statistical significance for the differences. The weight coefficient of each index was determined by establishing the judgement matrix using yaahp12.9 software (a software developed by Shanxi Yuanshi Software Technology Co., Ltd) with the Analytic Hierarchy Process (AHP).

### Quality control

To ensure the scientific credibility and accuracy of the research results, the criteria for selecting experts were strictly formulated. Garnett et al [27]claimed that choosing experts from the same background may contribute to a certain degree of bias in the results of a study. Accordingly, in this study, the experts we selected were devoted to multiple research areas. If the returned questionnaires were found to be incorrect or incomplete, we would contact the experts in time for verification. If more than 10% of the questionnaire was not completed, it would be considered as an invalid questionnaire and eventually deleted. All data were entered by two researchers using Excel 2010 software.

## Results

### Expert sociodemographic information

The present research enrolled a panel of 20 experts from Sichuan, Chongqing, Guangxi, Shanghai, Yunnan, Hubei, Guizhou, Hunan and Guangdong in China. The working years of experts are between 8 and 38 years. All the experts had experience working with an epidemic. The sociodemographic details of the experts were presented as Table 1.

**Table 1:** The socio-demographic information of the experts

Project	Round 1(n = 20)		Round 2(n = 18)	
	Frequency	Composition ratio	Frequency	Composition ratio
**Gender**				
male	10	50	10	55.56
female	10	50	8	44.44
**Age (years)**				
30~39	8	40	8	44.44
40~49	9	45	8	44.44
≥ 50	3	15	2	11.11
**Highest education**				
Undergraduate	5	25	4	22.22
Master	9	45	9	50.00
Doctor	6	30	5	27.78
**Professional positions**				
medium-grade professional position	8	40	8	44.44
Associate senior professional position	7	35	6	33.33
Senior professional position	5	25	4	22.22
**Specialist areas**				
public health management	4	20	4	22.22
infectious diseases prevention and control	5	25	4	22.22
disease control management	9	45	8	44.44
psychology	2	10	2	11.11
**Work experience (years)**				
8~15	8	40	8	44.44
16~23	5	25	3	16.67
≥ 24	7	35	7	38.89

### Enthusiasm and authority coefficient of experts

The enthusiasm of the experts can be expressed by the response rate of the questionnaire. It is generally considered that experts have fairly high enthusiasm when the response rate is greater than 70% [28]. In the first round, 20 questionnaires were distributed and all of them were returned, with a response rate of 100%. In the second round, 20 questionnaires were distributed, and 18 effective questionnaires were returned, with a response rate of 90% (Table 2). It is claimed by researchers that the expert consultation authority coefficient > 0.7 is considered to be reliable [29].Through rigorous calculation, the authority coefficient (Cr) of the two rounds of expert consultation were 0.88 and 0.90, suggesting that the experts were highly authoritative.

**Table 2:** Positive coefficient of experts

	Round 1	Round 2
**Questionnaire recovery**		
Total distribution(n)	20	20
Total response(n)	20	18
Response rate(%)	100	90
Effective proportion(%)	100	100
**Proposed ratio**		
Number of experts(n)	12	2
Constituent ratio(%)	60	11.1

### Degree of concentration and coordination of experts’ opinions

In the first round of consultation, the importance scores of all items ranged from 3.55 to 4.95, and the full mark rate was greater than 20%. The coefficient of variation (CV) was less than 0.25, except for one index whose coefficient of variation (CV) was 0.26. The Kendall’s W was 0.294 (χ^2^ = 470.614, *P* < 0.001). In the second round of consultation, the importance scores of all items ranged from 3.83 to 5.00. The full mark rate was greater than 20%, and the coefficient of variation (CV) was less than 0.25. The Kendall’s W was 0.322 (χ^2^ = 445.703, *P* < 0.001). (Table 3)

**Table 3:** The results of the degree of coordination among experts

Round	Hierarchical level	Items(n)	Kendall’s W	x^2^	*P*
Round 1	First-level	5	0.181	14.481	0.006
	s-level	21	0.246	98.591	<0.001
	Third-level	55	0.32	345.546	<0.001
	Total	81	0.294	470.614	<0.001
Round 2	First-level	5	0.185	13.343	0.01
	s-level	20	0.323	110.434	<0.001
	Third-level	53	0.322	301.362	<0.001
	Total	78	0.322	445.703	<0.001

### The formation process of the evaluation index system

In this study, we conducted two rounds of consultation. According to the screening criteria of indicators, experts’ opinions, and the research group discussion, the indicator system was adjusted until a consensus was reached (Table 4).

**Table 4:** The process of items revision

Round	Total number of items	Number of qualified items	Number of modified items	Number of deleted items	Number of added items	Number of merged items
Round 1	81	80	13	7	6	3
Round 2	78	78	4	0	0	0

In the first round of consultation, the experts did not make any comments on the first-level indicators, so they were all retained. However, the second-level indicators, three experts pointed out that ' cooperate with epidemic prevention and control of each department ' had logical problems with the same level indicators, they recommend revising it to ' cooperate with epidemic prevention and control of responsible departments '. The traffic health quarantine station is also a responsible department for epidemic prevention and control [21], experts suggested that it should be a third-level indicator, we deleted the ' cooperate with quarantine of department of transportation and health ' and adjusted it to the third-level indicators. While in the third-level indicators, several experts claimed that the public is not yet fully equipped to analyze the secondary disasters caused by MEIDs, and accordingly 'analysis of possible secondary disasters caused by epidemic' was deleted. Three experts pointed out that 'strictly comply with centralized isolation requirements ' was not clearly defined, and it should be revised to 'strictly comply with various control requirements related to isolation' which could make it more comprehensive. According to the experts’ suggestions, in addition to revising the above items, duplicate indicators were removed, and combined with the special requirements during MEIDs, the content of personal emergency preparedness was supplemented, as presented in Appendix2. Then, a new consultation questionnaire was developed and a total of 78 indicators were incorporated into the round 2 survey.

In the second round of expert consultation, experts had relatively unified opinions on each indicator. Only two experts proposed amendments, including adjusting the order of indicators and modifying the expression of indicators. In the reserve of emergency supplies during MEIDs, it was not recommended people to purchase unusually large amounts of products for which would result in a frenzied purchasing of supplies [30]. Thus, experts suggested changing ' purchase sufficient amount of emergency living goods, such as grain, oil and rice ' to ' purchase appropriate amount of emergency living goods, such as grain, oil and rice '. The other three similar indicators were also modified like this. Through the feedback of two rounds of Delphi study, the details of the content of the evaluation index system have been enriched and improved. All the experts agreed on the index system, the evaluation index system was finally formed, consisting of five first-level indicators, cooperating with prevention and control work, improving emergency response capacity, securing supplies and equipment, preparing economic resources, maintaining physical and mental health with affiliated 20 second-level indicators and 53 third-level indicators (Table 5).

### The index weight

AHP could analyze experts’ subjective judgment with mathematical form and conduct multi-objective decision-making analysis of scientific treatment to ensure the best results [26]. The weights of the indicators were calculated by the AHP according to the experts’ opinion, and the consistency test of all levels of indicators were CR < 0.10, demonstrating the judgment matrix was within the accepted range. The first-level indicators, cooperation with prevention and control work (0.3892) showed the highest value, followed by fully guaranteed supplies and equipment (0.2474), while improving emergency response capacity and maintaining physical and mental health showed the same value (0.1386). Finally, preparing economic resources (0.0862) was the smallest. The index weight of all levels of indicators are shown in Table 5.

**Table 5:** The emergency preparedness evaluation index system for public use during MEIDs

Indicators	Mean ± SD	CV	Full mark rate	weight
1.Cooperate with prevention and control work	4.89 ± 0.32	0.07	0.89	0.3892
1.1Cooperate with responsible department epidemic control	4.89 ± 0.32	0.07	0.89	0.0904
1.1.1Compliance with government policies and decrees on epidemic prevention and control	4.94 ± 0.24	0.05	0.94	0.0199
1.1.2Cooperate in implementing the programs and measures formulated by disease prevention and control institutions for epidemic prevention and control	4.89 ± 0.32	0.07	0.89	0.0113
1.1.3Go to medical institutions during the epidemic should follow their diagnosis and treatment procedures	4.83 ± 0.51	0.11	0.89	0.0081
1.1.4Cooperate with the epidemic situation control work in the place where the individual is located	4.94 ± 0.24	0.05	0.94	0.0199
1.1.5Cooperate with epidemic prevention and control at traffic stations	4.94 ± 0.24	0.05	0.94	0.0199
1.1.6Cooperate with epidemic prevention and control at entry-exit ports	4.89 ± 0.32	0.07	0.89	0.0113
1.2Cooperate with community epidemic prevention	4.94 ± 0.24	0.05	0.94	0.1538
1.2.1Proactively report personal status to the community where you go	5.00 ± 0.00	0.00	1.00	0.0638
1.2.2Cooperate with the collection and report of relevant personal information during epidemic situation	4.94 ± 0.24	0.05	0.94	0.0375
1.2.3Strictly comply with various control requirements related to isolation	4.94 ± 0.24	0.05	0.94	0.0375
1.2.4Understand and support possible omissions in epidemic prevention and control under limited conditions	4.67 ± 0.59	0.13	0.72	0.0151
1.3Cooperate with the work of mass prevention and control	4.83 ± 0.51	0.11	0.89	0.0547
1.3.1Strictly obey the disinfection prevention and control requirements	4.72 ± 0.46	0.10	0.72	0.0162
1.3.2Cooperate with the requisition of private property if necessary	4.06 ± 0.80	0.20	0.33	0.0038
1.3.3Understand the benefits of individual cooperation in prevention and control	4.22 ± 0.73	0.17	0.39	0.0069
1.3.4Cooperate with epidemic control in public places	4.78 ± 0.43	0.09	0.78	0.0228
1.3.5Actively participate in voluntary service for epidemic prevention and control	4.11 ± 0.83	0.20	0.39	0.0050
1.4Comply with laws and regulations	4.89 ± 0.32	0.07	0.89	0.0904
1.4.1Comply with infectious disease laws and regulations	4.83 ± 0.38	0.08	0.83	0.0251
1.4.2Do not fabricate or disseminate false epidemic information	4.72 ± 0.58	0.12	0.78	0.0148
1.4.3Do not conceal or forge personal information	4.94 ± 0.24	0.05	0.94	0.0357
1.4.4Do not hinder the staff to perform official duties	4.72 ± 0.46	0.10	0.72	0.0148
2.Improve emergency response capacity	4.61 ± 0.50	0.11	0.61	0.1386
2.1Learn the knowledge of infectious disease prevention and control	4.56 ± 0.62	0.14	0.61	0.0301
2.1.1Learn basic knowledge of infectious diseases	4.50 ± 0.62	0.14	0.56	0.0094
2.1.2Learn about the dangers of infectious diseases	4.39 ± 0.61	0.14	0.44	0.0059
2.1.3Learn about the prevention and control measures of infectious disease	4.61 ± 0.50	0.11	0.61	0.0149
2.2Identify the correct epidemic information	4.50 ± 0.62	0.14	0.56	0.0228
2.2.1Pay attention to the information related to epidemic situation released by authorities and departments	4.61 ± 0.50	0.11	0.61	0.0171
2.2.2Multi-channel verification of information content to improve the ability to distinguish the authenticity of epidemic information	4.33 ± 0.77	0.18	0.50	0.0057
2.3Adjuste risk perception	4.17 ± 0.86	0.21	0.39	0.0091
2.3.1Accurately confirm the possibility of self-infection	4.33 ± 0.59	0.14	0.39	0.0028
2.3.2Increase awareness of epidemic risk	4.50 ± 0.62	0.14	0.56	0.0045
2.3.3Pay attention to dynamic changes of epidemic	4.28 ± 0.58	0.13	0.33	0.0018
2.4 Improve protection capability	4.83 ± 0.51	0.11	0.89	0.0608
2.4.1Maintain good personal hygiene	4.83 ± 0.38	0.08	0.83	0.0072
2.4.2Maintain a safe social distance	4.89 ± 0.32	0.07	0.89	0.0123
2.4.3Do well in disinfection when going out and getting home	4.61 ± 0.61	0.13	0.67	0.0042
2.4.4Wear protective equipment correctly	4.94 ± 0.24	0.05	0.94	0.0177
2.4.5Take the initiative to monitor the health of family members and individuals	4.89 ± 0.32	0.07	0.89	0.0123
2.4.6Actively vaccinate the corresponding vaccine	4.83 ± 0.38	0.08	0.83	0.0072
2.5 Seek institutional help	4.33 ± 0.59	0.14	0.94	0.0157
2.5.1Know in advance the categories of organizations that can provide assistance	4.39 ± 0.50	0.11	0.39	0.0030
2.5.2 Be familiar with helplines of various institutions	4.28 ± 0.67	0.16	0.39	0.0019
2.5.3Familiar with the process of seeking help	4.44 ± 0.62	0.14	0.50	0.0043
2.5.4Understand emergency medical procedures	4.56 ± 0.62	0.14	0.61	0.0066
3.Fully guarantee supplies and equipment	4.78 ± 0.43	0.09	0.78	0.2474
3.1 Reserve protective equipment	4.50 ± 0.62	0.14	0.56	0.0857
3.1.1 Purchase appropriate amount of household protective equipment, such as masks, disposable gloves, etc.	4.67 ± 0.59	0.13	0.72	0.0857
3.2 Perfect the communication device	4.44 ± 0.71	0.16	0.56	0.0518
3.2.1Ensure that personal mobile phones or other means of communication are unobstructed	4.50 ± 0.62	0.14	0.56	0.0518
3.3 Understand the traffic situation	4.00 ± 0.69	0.17	0.22	0.0243
3.3.1Understand the traffic operation changes of individual travel route and destination	4.61 ± 0.50	0.11	0.67	0.0243
3.4Reserve emergency supplies	4.50 ± 0.51	0.11	0.50	0.0857
3.4.1Purchase appropriate amount of emergency living goods, such as grain, oil and rice	4.17 ± 0.79	0.19	0.44	0.0214
3.4.2Purchase appropriate amount of emergency medications, such as antipyretics	4.28 ± 0.75	0.18	0.44	0.0428
3.4.3Purchase appropriate amount of emergency tools, such as power supply equipment	4.17 ± 0.79	0.19	0.39	0.0214
4.Prepare economic resources	4.39 ± 0.70	0.16	0.50	0.0862
4.1 Estimate loss of income	3.89 ± 0.76	0.19	0.22	0.0104
4.1.1Estimate the loss of personal economic income caused by the epidemic	3.83 ± 0.86	0.22	0.28	0.0104
4.2 Estimate expenditure on epidemic prevention	4.11 ± 0.68	0.16	0.28	0.0281
4.2.1Estimate expenditure on purchasing epidemic prevention materials	3.89 ± 0.76	0.19	0.22	0.0281
4.3 Estimate medical expenditure	4.00 ± 0.69	0.17	0.22	0.0212
4.3.1Estimate personal medical expenses due to the epidemic	3.83 ± 0.79	0.21	0.22	0.0212
4.4 Estimate other expenditures	3.94 ± 0.80	0.20	0.22	0.0160
4.4.1Estimate personal expenses other than epidemic prevention and medical expenses, such as living expenses	3.78 ± 0.88	0.23	0.22	0.0160
4.5Adjusting the overall economy	3.89 ± 0.83	0.21	0.22	0.0104
4.5.1Adjust economic resources according to income and expenditure	3.94 ± 0.80	0.20	0.28	0.0104
5.Maintain physical and mental health	4.61 ± 0.61	0.13	0.67	0.1386
5.1 Maintain physical health	4.50 ± 0.51	0.11	0.50	0.0924
5.1.1Regular work and rest during epidemic situation	4.33 ± 0.84	0.19	0.56	0.0181
5.1.2Keep exercising	4.50 ± 0.71	0.16	0.61	0.0456
5.1.3Ensure a healthy diet	4.44 ± 0.78	0.18	0.61	0.0287
5.2 Maintain mental health	4.44 ± 0.62	0.14	0.50	0.0462
5.2.1Establish correct awareness of the epidemic and reduce undue panic	4.61 ± 0.61	0.13	0.72	0.0308
5.2.2Reasonably control personal emotions and seek psychological assistance if necessary	4.50 ± 0.62	0.14	0.56	0.0154

Note: Full mark rate = Total number of experts who gave 5 points to the importance of each item/Total number of experts who evaluated each item; SD = standard deviation

## Discussion

### Content analysis of the evaluation index system

Emergency preparedness, while not an arbitrary concept, is dependent on many factors [31]. This study has compiled a set of relatively comprehensive rating index system, including 53 items of knowledge, skills, emotion and behavior tendency about personal emergency response in five aspects.

#### Cooperate with prevention and control work

From the experts’ preference, cooperate with prevention and control work (0.3892) is particularly important and indispensable in the public emergency preparedness during MEIDs. It clearly divides the public emergency preparedness dimension from the most basic cooperation with the individual level of epidemic prevention and control, then to cooperate with the mass prevention and control, and finally rises to cooperate with the laws and regulations. This part aims to assess the public basic attitude and awareness of infectious disease prevention and control. Previous studies have identified attitudes as a key determinant of emergency preparedness [32, 33]. After the outbreaks, the impact is generally global. However, it cannot be ignored that the form of prevention and control is complex and changeable, the difficulty of prevention and control is huge, and the task of prevention and control is also very arduous [34]. Upholding the concept of self-discipline, the public cooperate with the government, relevant departments and agencies to carry out epidemic prevention and control work, which are both self-help and altruism [35, 36]. Such as complying with government or community arrangements and truthfully reporting infectious disease exposure history.

#### Fully guaranteed supplies and equipment

Fully guarantee supplies and equipment (0.2474) aims to assess the public could or could not reserve living materials during the epidemic, including a series of food, protective tools, etc., which are indispensable for human beings depend during the MEIDs [37, 38]. When a major emerging infectious disease is underway, we should wear protective equipment which can reduce the chance of being infected, and can also prevent patients or asymptomatic infected persons from spreading the virus in society [39]. During this period, if the public lacks medicine to deal with common diseases, they have to go out to see a doctor, thus increasing the probability of infection. Experts also considered reserving emergency supplies to be fairly important, but we should reserve them properly. Thus, they suggested changing the ' sufficient ' to ' appropriate ' in this part. It was reported that people have opted for conformity consumption to obtain a sense of belonging and security from the group during the infectious disease, thereby alleviating inner fear [40]. However, people often scramble to emergency supplies, which may lead to panic behavior and have a serious negative impact on public health management [30]. Therefore, it should be emphasized to guide the public behavior of purchasing emergency supplies.

#### Improve emergency response capacity

Improving emergency response capacity(0.1386) includes personal cognition of infectious diseases, judgment of epidemic risk, self-protection and seeking help. The purpose of this part is mainly to evaluate whether the public has a clear understanding of infectious diseases, and whether they can protect their own safety through themselves or others during the outbreaks. When the public own the knowledge of infectious diseases, they may not have a strong sense of panic and can make a more accurate judgment on their current situation based on their own experience. When encountering difficulties, they should know about how to seek help from institutions [41]. In China, some green channels for special groups are generally set up.

#### Maintain physical and mental health

Maintaining physical and mental health (0.1386) just to evaluate whether the public has a healthy lifestyle, which can also reflect whether they could positively and steadily live through the outbreaks. Along with the development of positive psychology, the guidance of positive emotions in public should be strengthened in MEIDs, and the development of positive psychological qualities should be emphasized to promote physical and mental health [42]. Maintaining good health could increase individual resistance, which in turn could reduce infections during MEIDs. Also, the public may experience psychological problems such as anxiety, depression, insomnia, post-traumatic stress disorder, and suicidal thoughts due to excessive stimulation and inability to cope with the epidemic [42].Then there would be secondary disasters in the mental, especially those on the epidemic prevalence areas, and the mental trauma is more serious [43, 44].Therefore, it is particularly necessary to maintain mental health before and after the outbreak of MEIDs.

#### Prepare economic resources

Preparing economic resources (0.0862) seems no obvious significance. However, during the COVID-19 pandemic, a highly representative viral disaster, some public suffered financial difficulties indeed. Thus, it is necessary for the public to have the facility to use their own resources reasonably. Judging from the scores of the expert panel, the overall score is indeed the lowest, which may be because experts consider that China’s medical security department provides positive treatment guarantee for MEIDs, which can largely relieve the worries of patients [45]. However, the daily expenses of the public are still a problem. When they are confined at home or cannot go out to earn money due to special epidemic conditions, how to control their own deposits also needs to be carefully considered.

### The profound meaning of the evaluation index system

The COVID-19 is responsible for millions of deaths globally [46, 47], and continues to demonstrate the risks and profound health impacts that result from infectious disease emergencies. The lockdown measures introduced led to the collapse of medical systems, the outbreak of economic crises, and serious social disorder in many countries [48]. MEIDs and concomitant pandemic measures are highly destructive, sudden, complex and uncertain, which seriously threaten the health and safety of the public. The preparedness of the public to cope with emergencies is growing importance [49]. Efficient emergency preparedness can not only help the public respond to MEIDs, but also alleviate the negative emotions towards MEIDs [50]. Brown KL has pointed out that the public preparedness research mostly focuses on general emergency preparedness rather than preparedness on specific hazards [31]. In the context of the increasing number of new infectious diseases [51], it is particularly necessary to construct a specific evaluation index system and to evaluate the emergency preparedness of public, which not only allows the public to know the aspects of their weaknesses in preparedness, but also facilitates relevant educators to provide targeted guidance to them.

### Reliability and scientific credibility of the evaluation index system

The emergency preparedness evaluation index system for public use during MEIDs constructed in this study is scientific, comprehensive and diversified with the following characteristics. Firstly, it is scientific and authoritative to some extent, since it was built based on the the mature and widely used global national-level emergency preparedness index framework. This index system was also built based on massive literature support [21, 22, 52–55]. Secondly, it is reliable due to qualified experts. In this study, 20 experts are influential and outstanding in the prevention and control of MEIDs. Moreover, they are from different regions and different departments, reducing area distribution bias to some extent. At the same time, the experts involved in this study specialized in multiple research areas, they could give targeted suggestions to this study, thus making the evaluation index system more scientific and reliable. Thirdly, the evaluation index system is systematic and comprehensive, since it does not only include the basic behavior and skills to deal with MEIDs, but also consider the comprehensive preparation for legal compliance, economic estimation, material reserves, physical and mental health. The evaluation content runs through the whole epidemic response process. Fourthly, experts all held intermediate and above titles, of which senior professional titles and above account for 60%. In addition, three-quarters of the experts were postgraduate students, indicating that the suggestions and comments made by them were based on rich theoretical knowledge and practical experience. The authority coefficients of this research were 0.88 and 0.90, which proved that the authority of the study was assured. The Kendall’s concordance coefficient of the two rounds of consultation were statistically significant, so it suggested again that the results of the evaluation index system were scientific and reliable.

## Conclusion

A valid, reliable and scientific evaluation index system was established through two rounds of expert consultation. This emergency preparedness index system, as a precursor form, will further lay the foundation for the formation of an assessment instrument and provide reference for future education and training of the public. However, the initially constructed index system was still limited to the theoretical framework, and a further study will implement empirical research to analyze the reliability and validity of the index to verify its practicality, applicability and feasibility.

## Electronic supplementary material

Below is the link to the electronic supplementary material.

Supplementary Material 1

Supplementary Material 2

## Data Availability

The data that support the findings of this study are available from the corresponding author upon reasonable request.

## Abbreviations

AHP: Analytic Hierarchy Process
COVID-19: Corona virus disease 2019
CV: Coefficient of variation
Kendall’s W: Kendall coefficient of concordance
MEIDs: Major emerging infectious diseases
SD: Standard deviation
