# Supplementary material for: Constructing an emergency preparedness evaluation index system for public use during major emerging infectious disease outbreaks: a Delphi study
Source: BMC Public Health. 2023 Jun 8;23:1109. doi: 10.1186/s12889-023-15980-6 (PMC10249543; doi:10.1186/s12889-023-15980-6)
Supplement: Supplementary file 1 — Supplementary Material 1 [file 12889_2023_15980_MOESM1_ESM.pdf]

The initial evaluation index system

first-level indicators	second-level indicators	third-level indicators
1.Cooperate with prevention and control work	1.1Cooperate with epidemic prevention and control of each department	1.1.1 Compliance with government policies and decrees on epidemic prevention and control<br>1.1.2 Cooperate with flow investigation of disease prevention and control institutions<br>1.1.3 Go to medical institutions during the epidemic should follow their diagnosis and treatment procedures<br>1.1.4 Cooperate with the epidemic control work of the unit where the individual is worked
	1.2 Cooperate with the management of floating population	1.2.1 Proactively report personal status to the community where you arrive<br>1.2.2 Obey the community epidemic prevention and control arrangements<br>1.2.3 Cooperate with epidemic control in public places
	1.3 Cooperate with quarantine of department of transportation and health	1.3.1 Cooperate with epidemic prevention and control at traffic stations<br>1.3.2 Cooperate with epidemic prevention and control at entry-exit ports
	1.4 Cooperate with the work of mass prevention and control	1.4.1 Cooperate with epidemic information collection and reporting<br>1.4.2 Strictly comply with centralized isolation requirements<br>1.4.3 Strictly obey the disinfection prevention and control requirements
	1.5 Comply with laws and regulations	1.5.1 Comply with infectious disease laws and regulations<br>1.5.2 Do not fabricate or disseminate false epidemic information<br>1.5.3 Do not conceal or forge personal information<br>1.5.4 Do not hinder the staff to perform official duties
2.Improve emergency response capacity	2.1 Learn the knowledge of infectious disease prevention and control	2.1.1 Learn the basic knowledge of the epidemic process of infectious diseases<br>2.1.2 Learn the common symptoms of infectious diseases<br>2.1.3 Learn about the dangers of infectious diseases<br>2.1.4 Learn about the prevention and control measures of infectious disease

	2.2 Identify the correct epidemic information	2.2.1Pay attention to the information related to epidemic situation released by authorities and departments<br>2.2.2 Multi-channel verification of information content to improve the ability to distinguish the authenticity of epidemic information<br>2.2.3 Pay attention to the judgment and opinions of epidemic prevention and control experts on the epidemic situation
	2.3 Adjuste risk perception	2.3.1 Accurately determine the possibility of self-infection<br>2.3.2 Increase awareness of epidemic risk<br>2.3.3 Analysis of possible secondary disasters caused by epidemic<br>2.3.4 Pay attention to the dynamic changes of epidemic information
	2.4 Improve protection capability	2.4.1 Wear protective equipment correctly<br>2.4.2 Maintain good personal hygiene<br>2.4.3 Maintain a safe social distance<br>2.4.4 Do well in disinfection when going out and home<br>2.4.5 Actively vaccinate the corresponding vaccine<br>2.4.6 Take the initiative to monitor the health of family members and individuals
	2.5 Seek institutional help	2.5.1 Know in advance the categories of organizations that can provide assistance<br>2.5.2 Be familiar with the help telephone numbers of various institutions<br>2.5.3 Familiar with the process of seeking help
3.Fully guarantee supplies and equipment	3.1Reserve protective equipment	3.1.1 Purchase sufficient quantities of household protective equipment, such as masks, disposable gloves, etc.
	3.2 Perfect the communication device	3.2.1 Keep your personal phone open<br>3.2.2 Make sure the mobile phone network is working properly
	3.3 Understand the traffic situation	3.3.1 Be familiar with the changes of various public transport routes<br>3.3.2 Understand the epidemic situation and epidemic control policies of travel routes and destinations
	3.4 Reserve emergency supplies	3.4.1 Purchase sufficient quantities of emergency living goods, such as grain, oil and rice<br>3.4.2 Purchase sufficient quantities of emergency medicine, except those covering up infectious diseases

		3.4.3 Purchase sufficient emergency tools, such as power supply equipment
4.Prepare economic resources	4.1 Estimate loss of income	4.1.1 Estimate the loss of personal economic income caused by the epidemic
	4.2 Estimate expenditure on epidemic prevention	4.2.1 Estimate expenditure on purchasing epidemic prevention materials
	4.3 Estimate medical expenditure	4.3.1 Estimate individual medical expenses due to illness
	4.4 Estimate other expenditures	4.4.1 Estimate personal expenses other than epidemic prevention and medical expenses, such as living expenses
	4.5 Adjusting the overall economy	4.5.1 Adjust economic resources according to income and expenditure
5.Maintain physical and mental health	5.1 Maintain physical health	5.1.1 Regular work and rest during epidemic period
		5.1.2 Insist on regular exercise
		5.1.3 Ensure a healthy diet structure
	5.2 Maintain mental health	5.2.1 Establish correct understanding of the epidemic
		5.2.2 Actively ease negative emotions
		5.2.3 Actively establish positive emotions
