# Supplementary material for: Constructing an emergency preparedness evaluation index system for public use during major emerging infectious disease outbreaks: a Delphi study
Source: BMC Public Health. 2023 Jun 8;23:1109. doi: 10.1186/s12889-023-15980-6 (PMC10249543; doi:10.1186/s12889-023-15980-6)
Supplement: Supplementary file 2 — Supplementary Material 2 [file 12889_2023_15980_MOESM2_ESM.docx]

Appendix2: The revision of the third-level indicators of the evaluation index system

| Initial items | Outcome | Final items |
| --- | --- | --- |
| Cooperate with flow investigation of disease prevention and control institutions | modified | Cooperate in implementing the programs and measures formulated by disease prevention and control institutions for epidemic prevention and control |
| Cooperate with the epidemic control work of the unit where the individual is worked |  | Cooperate with the epidemic situation control work in the place where the individual is located |
| Cooperate with epidemic information collection and reporting |  | Cooperate with the collection and report of relevant personal information during epidemic situation |
| Strictly comply with centralized isolation requirements |  | Strictly comply with various control requirements related to isolation |
| Pay attention to the dynamic changes of epidemic information |  | Pay attention to dynamic changes of epidemic |
| Familiar with all kinds of agencies for help calls |  | Be familiar with the help telephone numbers of various institutions |
| Estimate individual medical expenses due to illness |  | Estimate personal medical expenses due to the epidemic |
| Insist on regular exercise |  | Keep exercising |
| Ensure a healthy diet structure |  | Ensure a healthy diet |
| Establish correct understanding of the epidemic |  | Establish correct awareness of the epidemic and reduce undue panic |
| 'Learning the basics of the epidemic process of infectious diseases ' and ' learning common symptoms of infectious diseases' | merged | Learn basic knowledge of infectious diseases |
| ' Keep your phone open ' and ' keep your mobile network working ' |  | Ensure that personal mobile phones or other means of communication are unobstructed |
| 'Positively channel negative emotions ' and ' correctly establish positive emotions ' |  | Reasonably control personal emotions and seek psychological assistance if necessary |
| Obey the community epidemic prevention and control arrangements | deleted |  |
| Pay attention to the Judgment and opinions of epidemic prevention and control experts on the epidemic |  |  |
| Be familiar with the changes of various public transport routes |  |  |
| Analysis of possible secondary disasters caused by epidemic |  |  |
|  | added | Understand and support possible omissions in epidemic prevention and control under limited conditions |
|  |  | Cooperate with the requisition of private property if necessary |
|  |  | Understand the benefits of individual cooperation in prevention and control |
|  |  | Actively participate in voluntary service for epidemic prevention and control |
|  |  | Understand emergency medical procedures |
